# Case Report: Genetic analysis of oculocutaneous albinism type 2 caused by a new mutation in the *OCA2*

**DOI:** 10.3389/fped.2025.1508198

**Published:** 2025-04-17

**Authors:** Lei Luo, Min Ma, Yanzhang Yang, Hui Zhao

**Affiliations:** ^1^ Department of Pediatrics and Neonatal, Hebei General Hospital, Shijiazhuang, China; ^2^ Department of Internal Medicine, The Fourth Hospital of Shijiazhuang (Maternal Hospital Affiliated to Hebei Medical University), Shijiazhuang, China; ^3^ Department of Pediatrics, The Second Hospital of Hebei Medical University, Shijiazhuang, China

**Keywords:** oculocutaneous albinism type 2, *OCA2*, new mutation site, WES (whole exome sequencing), case report

## Abstract

Oculocutaneous albinism (OCA) is a condition inherited in an autosomal recessive manner, leading to reduced pigmentation in the skin, hair, and eyes. Oculocutaneous albinism type 2 (OCA2) is one of the most common forms of OCA, caused by *OCA2* mutations. This case report presents a newborn with suspected OCA. Postnatal examination revealed white skin, golden-colored hair, and reduced visibility of the retinal pigmented epithelium on fundus photography. Genomic DNA was extracted from the peripheral blood of the patient and her parents. Whole Exome Sequencing (WES) was conducted using chip capture-based high-throughput sequencing technology to analyze genomic DNA from the proband and her parents. Genetic variants of his parents were identified using sanger sequencing. A mutation in the *OCA2* was identified: NM_000275.2: c.863_886delTGAGCAGGACCTTTGAGGTGA (p.Met288_Leu295del). Subsequently genetic analyses were conducted. This mutation was recognized as a potential disease-causing mutation, validating diagnosis of OCA2. Currently, few reports have been published regarding this mutation site. It represents a new mutation site in *OCA2* (NM_000275.2:c.863_886del), contributing to the genetic diversity of the *OCA2*.

## Introduction

Albinism is a genetic disorder caused by mutations in specific genes related to pigment production, resulting in the lack of melanin (1). It is inherited in an autosomal recessive manner (1). The prevalence of albinism is 5–10 per 100,000 in the general population. The prevalence is as high as 1 in 1,400 in some African countries. In contrast, the prevalence of albinism in China is approximately 1 in 18,000 (2). Albinism represents a genetically heterogeneous group of disorders characterized by impaired melanin biosynthesis. Contemporary classification divides albinism into two broad categories: syndromic albinism, which involves multi-system manifestations [e.g., Hermansky-Pudlak syndrome [HPS] with bleeding diathesis and pulmonary fibrosis, and Chediak-Higashi syndrome [CHS] with immune dysfunction], and non-syndromic albinism, comprising oculocutaneous albinism (OCA1-8) and ocular albinism (OA), primarily affecting pigmentation in the eyes, skin, and hair (1, 3, 4). This refined classification underscores the molecular diversity and clinical complexity of albinism.

*T*OCA is divided into 8 subtypes (OCA1-8) based on the pathogenic genes (1). OCA2 is the most prevalent form worldwide (5). OCA2 exhibits variable clinical features, characterized by mild-to-moderate hypopigmentation of the hair, skin, and iris. Notably, pigmentation levels often increase with age; thus, OCA2 has been classified as incomplete albinism (6). This dynamic phenotypic progression underscores the unique molecular pathogenesis of OCA2, where residual melanin synthesis may persist over time despite underlying genetic defects. OCA2 is specifically caused by mutations in the *OCA2* gene. There are different mutations of the *OCA2*, including missense, deletion, and frameshift mutations (7). In this study, WES was conducted on a newborn with suspected albinism. A novel mutation of the *OCA2* was identified, confirming the diagnosis of OCA2.

### Case description

**Figure 1:**
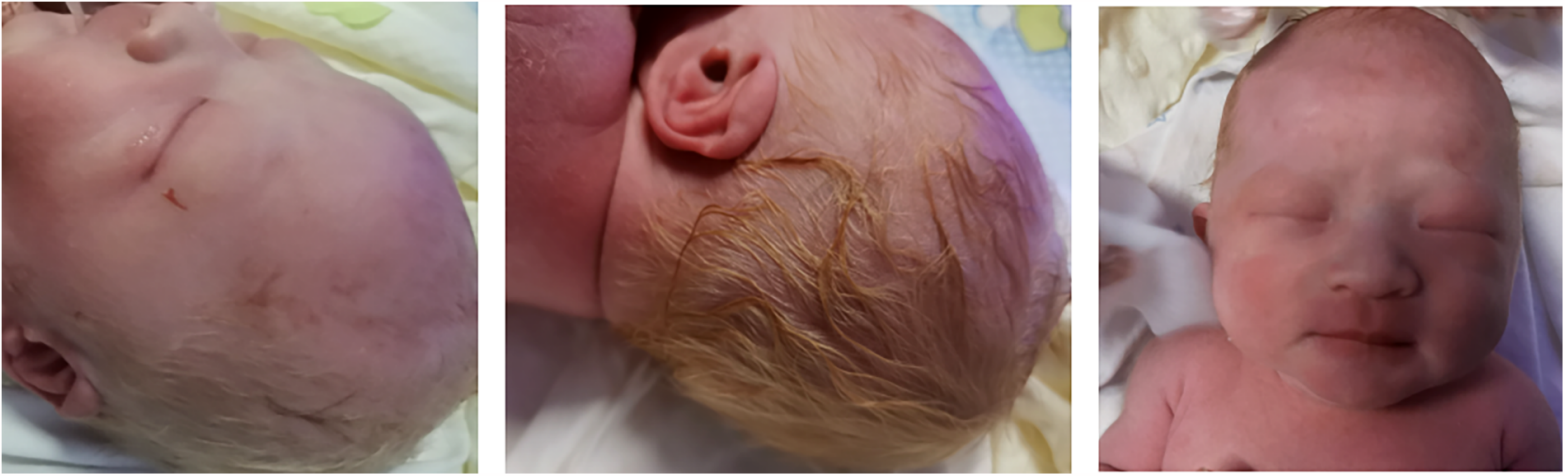
The proband had pink white skin and light and golden hair.

**Figure 2:**
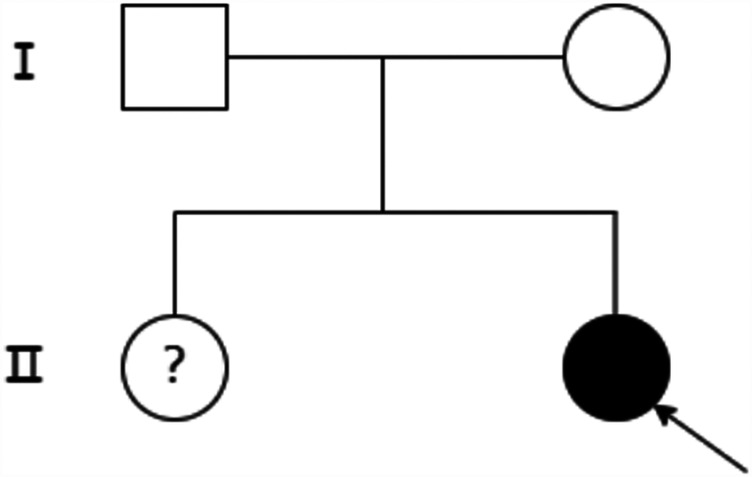
Family diagram of the proband. Black arrows indicate the proband, circles indicate female, boxes indicate male, hollow areas indicate normal individuals, and solid areas indicate OCA2 patients. The question mark (?) indicates undetermined genotype due to lack of genetic testing.

**Figure 3:**
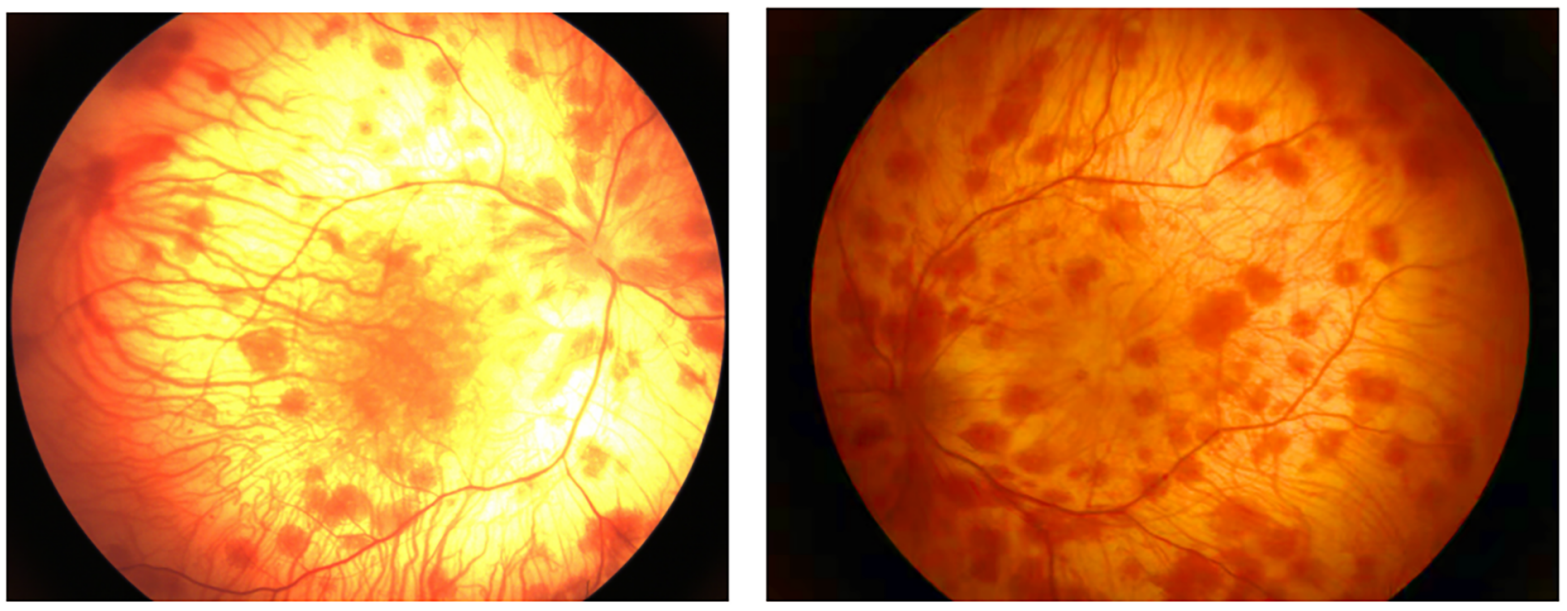
Fundus examination image. Extensive and light pigment in the retina, a large amount of patchy hemorrhage in the retina, and light pigment in the macula.

**Figure 4:**
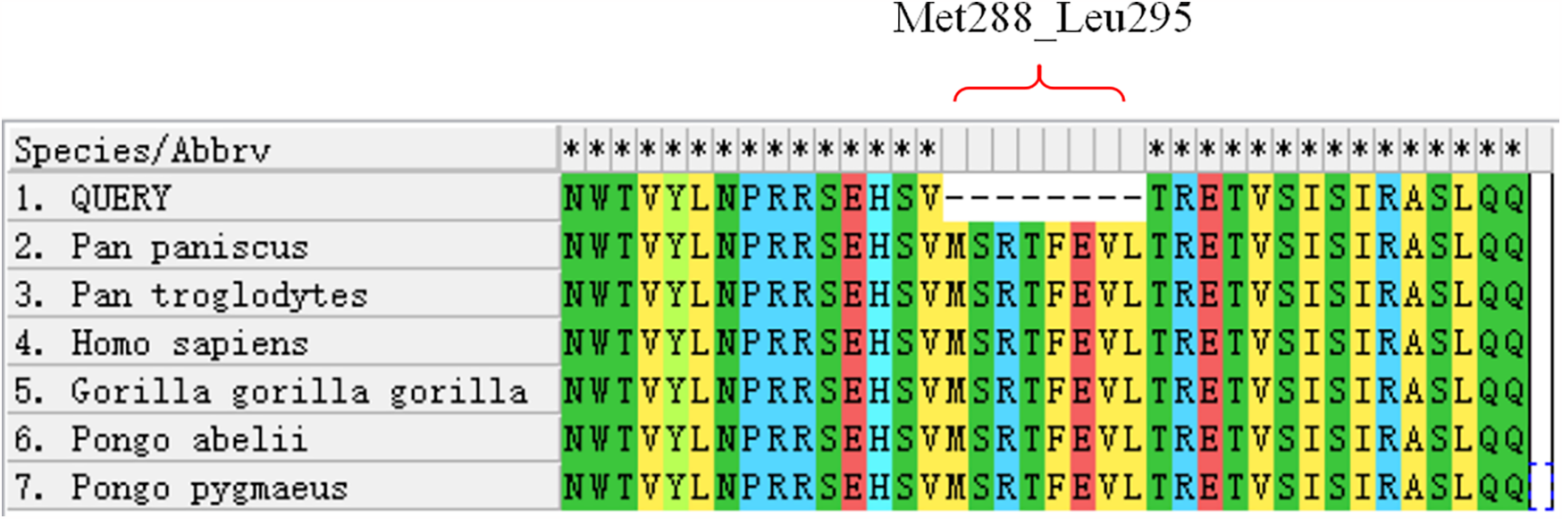
Conservation analysis of the OCA2 protein region (Met288-Leu295) across multiple species. The red bracket indicates the missing amino acid sequence in this region.

**Figure 5:**
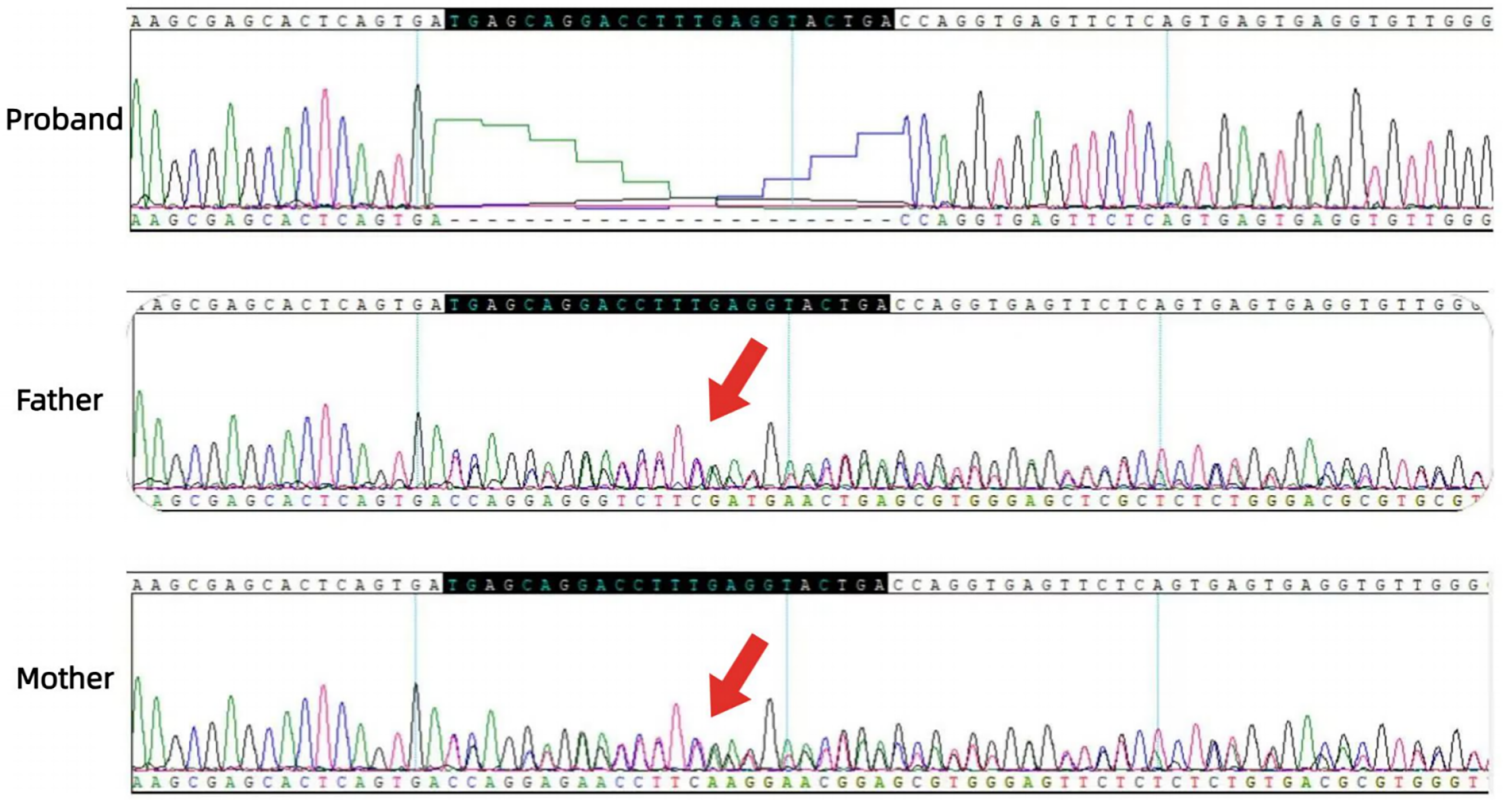
Sanger sequencing chromatograms of the OCA2 deletion (c.863_886del). The proband shows a distinct sequence shift (red bracket) from homozygous deletion. Top reference sequence alignment highlights the 24-bp deletion in the proband vs. wild-type. Parents have overlapping peaks (arrows) at the site, indicating heterozygous carriage.

A. Clinical presentation A female proband was admitted to our hospital 28 min after birth because of light-colored hair and skin. The baby was born at 38 ^+ 3^ weeks of gestation. The baby was the second child and second delivery of her mother, delivered vaginally with a bat-placenta. The umbilical cord or amniotic fluid exhibited no abnormalities, and the Apgar score was 10. After birth, the baby's hair and eyebrows had light, golden yellow color, and the skin was pinkish white (Figure 1). She had difficulty opening her eyes, had photophobia, and showed no nystagmus. The parents and the elder sister of the proband exhibited no clinical abnormalities, and there was no consanguinity between the parents (Figure 2). A thorough examination showed no abnormalities in peripheral blood cells, and liver function, kidney function, blood lipids, and electrolyte levels were all normal. Echocardiography showed no abnormalities. Blood and urine screening for genetic metabolic diseases revealed no hereditary mutations. Ophthalmic examination using the RetCam III system exhibited no abnormalities in the anterior segment of both eyes, lighter iris pigmentation, clear optic disc margins with normal color, and widespread light pigmentation in the retina (Figure 3). The retina displayed numerous scattered hemorrhages, but the vascular structure appeared healthy, showing complete development alongside slight pigmentation in the macula. The baby's skin and hair color, and the light retinal pigmentation highly suggested oculocutaneous albinism. WES was conducted to identify the pathogenic gene.
B. Genomic DNA extraction Peripheral blood samples were collected from the proband and her parents. Genomic DNA was isolated using the QIAamp DNA Blood Mini Kit (Qiagen, Germany). DNA purity and concentration were assessed via NanoDrop (A260/A280 ratio: 1.9) and Qubit 4.0 Fluorometer (Thermo Fisher Scientific, USA).
C. Library preparation DNA libraries were constructed using the Roche KAPA HyperExome Kit (Roche, Switzerland). Fragmented DNA underwent end repair, adapter ligation, and PCR amplification. Library quality was verified using an Agilent 2100 Bioanalyzer (Agilent Technologies, USA), ensuring fragment sizes of 200–300 bp.
D. Whole Exome Sequencing WES was performed on the MGISEQ-2000 platform (MGI Tech, China) with 150-bp paired-end reads. Target regions achieved an average depth of ≥200×, with >98.5% of bases covered at ≥20×.
E. Bioinformatic analysis Raw reads were aligned to the UCSC hg19 reference genome using BWA-MEM (v0.7.17). Duplicate reads were removed with Picard Tools (v2.27.5). SNVs and INDELs were identified using GATK HaplotypeCaller (v4.2.6.1). ExomeDepth (v1.1.16) was employed for exon-level CNV detection.
F. Variant identification Variants were filtered against GnomAD, ExAC, 1000 Genomes Project, and dbSNP to exclude benign polymorphisms. A novel deletion in *OCA2* (NM_000275.2:c.863_886del) was absent in all public databases. Bioinformatic prediction analyses using Mutation Taster, PROVEAN and SIFT indicated that they were deleterious. Amino acid sequences upstream and downstream of the gene mutation site were selected from different species, and the conservation of the encoded protein sequence among different species was analyzed using MEGA11 software. The results showed that the site was highly conserved among species (Figure 4). The mutations at highly conserved sites also confirmed its potential pathogenicity.
G. Sanger sequencing and segregation Analysis The primers were designed as follows: *OCA2*_Ex8F (forward primer, 5′-TCCAACCACCTGCACTAGCTC-3′) and *OCA2*_Ex8R (reverse primer, 5′-TACGATGTCCTCCCACAGCCT-3′). Amplification was conducted with annealing temperatures of 60.1°C for the forward primer and 61.7°C for the reverse primer, yielding a 396-bp product spanning the targeted region of *OCA2* exon 8. In Figure 5, the chromatogram revealed heterozygous peaks (double peaks) at the mutation site (chr15:28,261,254 - 28,261,277) in both parents, which is indicative of the presence of both wild-type and deleted alleles, confirming their status as heterozygous carriers of the deletion. In contrast, the proband's chromatogram displayed a distinct frameshift pattern with no overlapping peaks, confirming the absence of the wild-type allele and the homozygous nature of the deletion. Alignment of sequencing results with the reference *OCA2* sequence (NM_000275.2) confirmed a 24-bp deletion (c.863_886del), which corresponds to an in-frame removal of eight amino acids (p.Met288_Leu295del). The pedigree confirmed autosomal recessive inheritance.
H. Variant classification (ACMG/AMP Guidelines) The variant was classified as likely pathogenic (PM2 + PM3_Supporting + PM4 + PP4). PM2: Absent in population databases. PM3_Supporting: Biallelic inheritance confirmed. PM4: In-frame deletion altering protein length. PP4: Phenotype consistent with OCA2.

Follow up At 7 months, the infant demonstrated normal motor development (rolling, object transfer) but no remarkable improvement in pigmentation. Sun protection measures were emphasized.

Ethical approval This study was approved by the Ethics Committee of Hebei General Hospital (Approval NO.2024-LW-0183) and conducted in accordance with the Declaration of Helsinki (2013). Informed consent was obtained from the parents.

## Discussion

Melanocytes are widely distributed in several organs, such as the skin and eyes. They can synthesize and release melanin to reduce ultraviolet damage. Melanin is synthesized in melanosomes, and its maturation occurs through four stages. Stages I and II are premelanosomes. Melanin synthesis and accumulation begin in stage III. Melanin accumulation will be complete in stage IV (8–10). Albinism is a group of hereditary disorders caused by mutations of genes involved in melanin synthesis, leading to the lack of melanin in the eyes, skin, and hair. Affected individuals have lighter or no color in the corresponding areas. The skin lacks pigment protection, making it sensitive to ultraviolet and prone to skin cancer. Patients with albinism suffer from photophobia but have normal night vision. They may also suffer from decreased visual acuity, have underdeveloped fovea, exhibit changes in the optic chiasma, and experience nystagmus. The OCA2 phenotype is caused by mutations of the *OCA2* (also known as the *P* gene). It is located on chromosome 15q11.2-q12, and includes 25 exons and 24 introns, spanning 345 kb in length. The protein translation starts from the second exon, encoding a transmembrane protein composed of 838 amino acid residues with a molecular weight of 10 kDa, functioning as a transporter on the melanosome membrane (11). The OCA2 protein has 12 transmembrane domains (12) and 3 glycosylation sites (13), participating in the transportation of tyrosine within melanocytes. It is a precursor for melanin synthesis. The P protein can regulate the pH of melanosomes and control melanosome maturation, serving as a key checkpoint for determining human skin color (14–16). In this study, the mutation of the *OCA2* occurred at EX8/CDS7, leading to a deletion mutation in the encoded protein (Met288_Leu295del). This mutation site has not been previously reported and represents a novel mutation site. Compared to the normal melanosome membrane protein, the mutant protein lacks 8 amino acids, and the harmfulness prediction suggested it was a harmful mutation. The site was highly conserved across species. Mutations at highly conserved sites also increase disease risk, leading to abnormal protein function, abnormal tyrosine transportation, inhibition of melanin formation, and albinism.

OCA2 is more common in Africans and African Americans, with an incidence of 1/37,000 in Caucasians, 1/3,900 in Bantu-speaking people in southern Africa, and 1/15,000 in African Americans (17). The clinical manifestations of OCA2 are relatively mild and affected individuals have light yellow hair at birth due to mild-to-moderate pigmentation, which may increase with age; however, skin pigmentation generally does not increase with sun exposure. The affected patients may also suffer from nystagmus, strabismus, and decreased visual sensitivity due to abnormalities in the visual nerve conduction pathway. The phenotypic variation of OCA2 is wide. Brown OCA may also occur, mainly in people of South African descent (18). A molecular epidemiological survey of 179 patients with albinism by Chinese scholars showed that OCA2 accounts for 11.7% of albinism in China (19). The *OCA2* regulates eye color mainly via the upstream gene HERC2. The key site rs-12913832 directly affects the expression of P protein, thereby controlling melanin synthesis in the human eye and regulating eye color (20–23). This child was of Asian descent, with pinkish-white skin after birth, mild pigment deficiency, and golden yellow hair. It is difficult to diagnose albinism solely based on clinical presentation. A complete fundus examination indicated widespread lightening of iris pigment, retinal pigment, and macular pigment. Subsequent WES gene testing identified the mutation of the *OCA2*. Sanger validation of the mutation site in the parents of the proband showed that each parent had one chromosome with a deletion mutation, both heterozygous with no clinical phenotype. The proband inherited the mutated chromosome from both parents, becoming homozygous and suggesting that c.863_886del is pathogenic. It is also necessary to be vigilant for Prader-Willi syndrome (PWS) and Angelman syndrome (AS) when observing reduced pigmentation in the skin and hair of children. PWS is a complex genetic disorder affecting several systems. It is caused by the lack of expression of paternal genes in the 15q11.2-q13 region. PWS presents with typical facial features: narrow forehead, almond-shaped eyes, narrow nose, thin upper lip, and downturned corners of the mouth, difficulty feeding in infancy, obesity in childhood, delayed development, cognitive impairment, and poor gonadal development (24). The main cause of AS is the abnormality of maternal genes in the 15q11-13 region. The main clinical features of AS include intellectual disability, motor disorders, language delay, abnormal gait, happy behavior, microcephaly, hyperactivity, epilepsy, and abnormal electroencephalogram (25). The *OCA2* is also located in the 15q11-q13 region. Studies have found that the *OCA2* in patients with PWS or AS is hemizygous, and due to the loss of the missing segment of the *OCA2*, patients with PWS or AS usually exhibit hypopigmentation (26). Comorbidity occurs after the deletion of paternal or maternal alleles in the 15q11-q13 region of chromosome 15; therefore, some patients with PWS and AS exhibit the clinical manifestations of albinism (27). Albinism usually does not affect life expectancy, development, intelligence, and fertility, but photosensitivity limits the outdoor activities of patients. Treatment alleviates the symptoms, improves the quality of life, and decreases the risk of complications. This child had no specific facial features and was followed for 7 months after birth, showing normal development. It is necessary to be vigilant for other comorbidities among children with delayed development.

## Conclusion

This study analyzed the mutation sites and phenotypes of *OCA2* in the proband and her family. The novel *OCA2* deletion mutation, NM_000275.2:c.863_886del TGAGCAGGACCTTTGAGGTACTGA(p.Met288_Leu295del), reported for the first time in this study, expanded the mutation spectrum of the *OCA2*. These findings provide a basis for future research on the role of OCA2 and genotype-phenotype correlations and strengthen clinicians’ understanding of the disease.

## Data Availability

The original contributions presented in the study are included in the article/Supplementary Material, further inquiries can be directed to the corresponding author.
